# Bis(acetyl­acetonato-κ^2^ *O*,*O*′)(pyridine-κ*N*)(thio­cyanato-κ*N*)manganese(III): a redetermination using data from a single crystal

**DOI:** 10.1107/S1600536813030407

**Published:** 2013-11-13

**Authors:** Stefan Suckert, Inke Jess, Christian Näther

**Affiliations:** a Institut für Anorganische Chemie, Christian-Albrechts-Universität Kiel, Max-Eyth-Strasse 2, 24118 Kiel, Germany

## Abstract

In the crystal structure of the title compound, [Mn(C_5_H_7_O_2_)_2_(NCS)(C_5_H_5_N)], the Mn^3+^ cation is coordin­ated by two acetyl­acetonate anions, one terminal thio­cyanate anion and one pyridine ligand within a slightly distorted octa­hedron. The asymmetric unit consists of half a complex mol­ecule with the Mn^3+^ cation, the thio­cyanate anion and the pyridine ligand located on a mirror plane. The acetyl­acetonate anion is in a general position. The title compound was previously described [Stults *et al.* (1975 ▶). *Inorg. Chem.* **14**, 722–730] but could only be obtained as a powder. Suitable crystals have now been obtained for a high-precision single-crystal structure determination.

## Related literature

For the preparation of the title compound in the form of a powder, see: Stults *et al.* (1975 ▶).
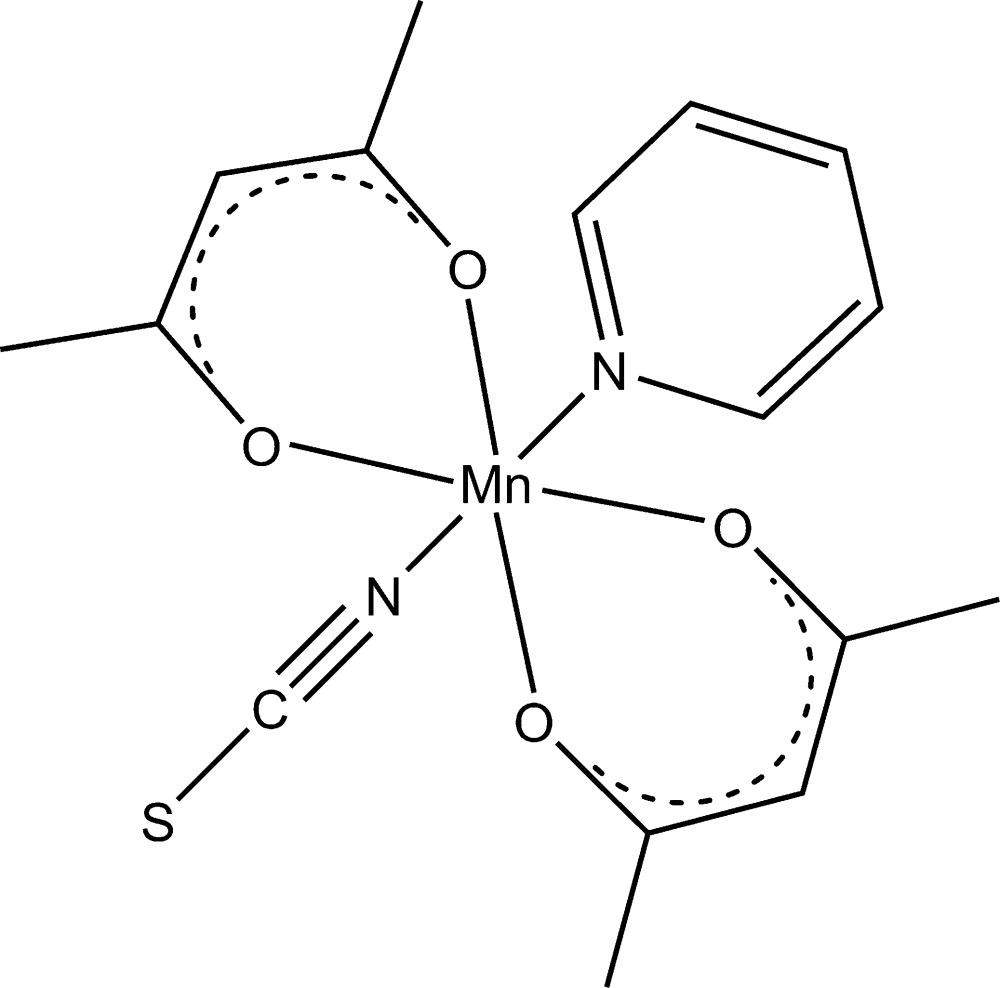


## Experimental

### 

#### Crystal data

[Mn(C_5_H_7_O_2_)_2_(NCS)(C_5_H_5_N)]
*M* *_r_* = 390.33
Orthorhombic, 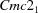

*a* = 13.8803 (6) Å
*b* = 8.3195 (5) Å
*c* = 15.9035 (7) Å
*V* = 1836.49 (16) Å^3^
*Z* = 4
Mo *K*α radiation
μ = 0.85 mm^−1^
*T* = 200 K
0.17 × 0.14 × 0.09 mm

#### Data collection

STOE IPDS-2 diffractometer
Absorption correction: numerical (*X-SHAPE* and *X-RED32*; Stoe & Cie, 2008 ▶) *T*_min_ = 0.802, *T*_max_ = 0.883
6523 measured reflections
2267 independent reflections
2090 reflections with *I* > 2σ(*I*)
*R*_int_ = 0.028

#### Refinement

*R*[*F* ^2^ > 2σ(*F* ^2^)] = 0.030
*wR*(*F* ^2^) = 0.066
*S* = 1.04
2267 reflections
126 parameters
1 restraint
H-atom parameters constrained
Δρ_max_ = 0.22 e Å^−3^
Δρ_min_ = −0.18 e Å^−3^
Absolute structure: Flack (1983 ▶), 1086 Friedel pairs
Absolute structure parameter: 0.015 (19)

### 

Data collection: *X-AREA* (Stoe & Cie, 2008 ▶); cell refinement: *X-AREA*; data reduction: *X-AREA*; program(s) used to solve structure: *SHELXS97* (Sheldrick, 2008 ▶); program(s) used to refine structure: *SHELXL97* (Sheldrick, 2008 ▶); molecular graphics: *XP* in *SHELXTL* (Sheldrick, 2008 ▶); software used to prepare material for publication: *SHELXL97* and *publCIF* (Westrip, 2010 ▶).

## Supplementary Material

Crystal structure: contains datablock(s) I, global. DOI: 10.1107/S1600536813030407/bt6942sup1.cif

Structure factors: contains datablock(s) I. DOI: 10.1107/S1600536813030407/bt6942Isup2.hkl

Additional supplementary materials:  crystallographic information; 3D view; checkCIF report

## Figures and Tables

**Fig. 1.**
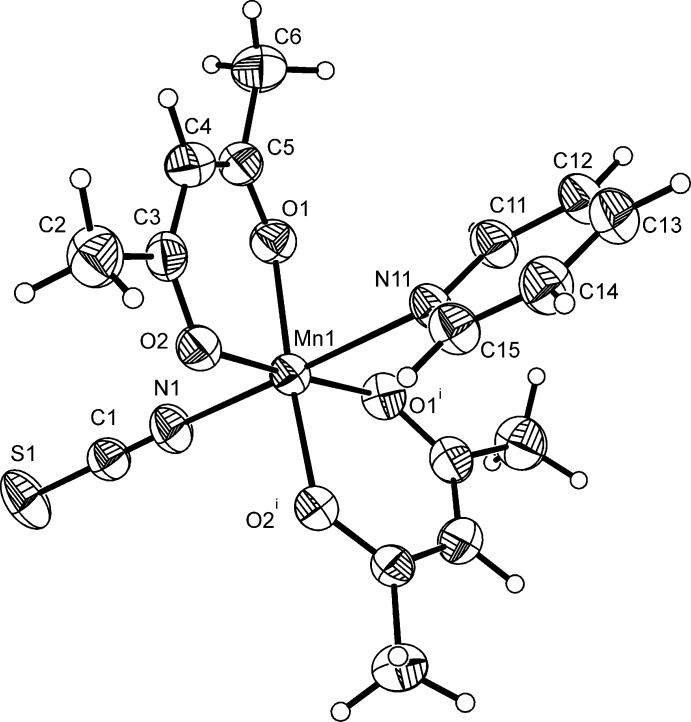
Crystal structure of the title compound with labelling and displacement ellipsoids drawn at the 50% probability level. Symmetry code: i = -x+1, y, z.

## supplementary crystallographic information

### 1. Comment

Crystals of the title compound were prepared within a project on the synthesis of Manganese(III) coordination polymers containing thiocyanato anions and neutral N-donor co-ligands. Within this project manganese(III) acetylacetonate was reacted with potassium thiocyanate and pyridine in a mixture of ethanol and sulfuric acid leading to the formation of crystals of the title compound. The title compound was already described by Stults *et al.* (1975) but could only be obtained as a microcrystaline powder. We now have been able to get suitable crystals for a single crystal structure determination.

In the crystal structure the manganese(III) cations are coordinated by four oxygen atoms of two symmetry related acetylacetone anions and two nitrogen atoms of an N-terminal coordinated thiocyanato anion and one pyridine ligand into discrete complexes that are located on a mirror plane (Fig. 1). The coordination polyhedron of the Mn cation can be described as a slightly distorted octahedron.

### 2. Experimental

Manganese(III) 2,4-pentadionate was purchased from Alfa Aesar. Potassium thiocyanate was purchased from Fluka. Pyridine was purchased from Riedel-de Haen. The title compound was prepared by the reaction of 70.5 mg Mn(III) 2,4-pentadionate (0.20 mmol) 58.3 mg potassium thiocyanate (0.6 mmol) and 32.3 µl pyridine (0.4 mmol) in a mixture of 1.0 mL ethanol and 10.68 µl sulfuric acid at RT in a closed 3 ml snap cap vial. After three days brown crystals of the title compound, mostly in the form of plates, were obtained by slow evaporation of the solvent.

### 3. Refinement

H atoms were positioned with idealized geometry (methyl H atoms allowed to rotate but not to tip) and were refined isotropically with *U*_eq_(H) = 1.2 *U*_eq_(C) for aromatic H atoms (1.5 for methyl H atoms) using a riding model with C—H = 0.95 Å (aromatic) and with C—H = 0.98 Å (methyl).

### Crystal data

**Table:**

[Mn(C_5_H_7_O_2_)_2_(NCS)(C_5_H_5_N)]	*F*(000) = 808
*M**_r_* = 390.33	*D*_x_ = 1.412 Mg m^−^^3^
Orthorhombic, *C**m**c*2_1_	Mo *K*α radiation, λ = 0.71073 Å
Hall symbol: C 2c -2	Cell parameters from 6523 reflections
*a* = 13.8803 (6) Å	θ = 2.9–28.0°
*b* = 8.3195 (5) Å	µ = 0.85 mm^−^^1^
*c* = 15.9035 (7) Å	*T* = 200 K
*V* = 1836.49 (16) Å^3^	Plate, brown
*Z* = 4	0.17 × 0.14 × 0.09 mm

### Data collection

**Table:**

STOE IPDS-2 diffractometer	2267 independent reflections
Radiation source: fine-focus sealed tube	2090 reflections with *I* > 2σ(*I*)
Graphite monochromator	*R*_int_ = 0.028
ω scan	θ_max_ = 28.0°, θ_min_ = 2.9°
Absorption correction: numerical (*X-SHAPE* and *X-RED32*; Stoe & Cie, 2008)	*h* = −16→18
*T*_min_ = 0.802, *T*_max_ = 0.883	*k* = −10→10
6523 measured reflections	*l* = −20→20

### Refinement

**Table:**

Refinement on *F*^2^	Secondary atom site location: difference Fourier map
Least-squares matrix: full	Hydrogen site location: inferred from neighbouring sites
*R*[*F*^2^ > 2σ(*F*^2^)] = 0.030	H-atom parameters constrained
*wR*(*F*^2^) = 0.066	*w* = 1/[σ^2^(*F*_o_^2^) + (0.0294*P*)^2^ + 0.9669*P*] where *P* = (*F*_o_^2^ + 2*F*_c_^2^)/3
*S* = 1.04	(Δ/σ)_max_ < 0.001
2267 reflections	Δρ_max_ = 0.22 e Å^−^^3^
126 parameters	Δρ_min_ = −0.18 e Å^−^^3^
1 restraint	Absolute structure: Flack (1983), 1086 Friedel pairs
Primary atom site location: structure-invariant direct methods	Absolute structure parameter: 0.015 (19)

### Special details

**Table:**

Geometry. All esds (except the esd in the dihedral angle between two l.s. planes) are estimated using the full covariance matrix. The cell esds are taken into account individually in the estimation of esds in distances, angles and torsion angles; correlations between esds in cell parameters are only used when they are defined by crystal symmetry. An approximate (isotropic) treatment of cell esds is used for estimating esds involving l.s. planes.
Refinement. Refinement of F^2^ against ALL reflections. The weighted R-factor wR and goodness of fit S are based on F^2^, conventional R-factors R are based on F, with F set to zero for negative F^2^. The threshold expression of F^2^ > 2sigma(F^2^) is used only for calculating R-factors(gt) etc. and is not relevant to the choice of reflections for refinement. R-factors based on F^2^ are statistically about twice as large as those based on F, and R- factors based on ALL data will be even larger.

### Fractional atomic coordinates and isotropic or equivalent isotropic displacement parameters (Å^2^)

**Table:**

	*x*	*y*	*z*	*U*_iso_*/*U*_eq_	
Mn1	0.5000	0.70123 (5)	0.26659 (3)	0.03145 (11)	
S1	0.5000	1.19178 (13)	0.09102 (7)	0.0591 (3)	
C1	0.5000	1.0340 (4)	0.1504 (2)	0.0365 (6)	
N1	0.5000	0.9206 (3)	0.19339 (18)	0.0442 (7)	
N11	0.5000	0.4703 (3)	0.35186 (15)	0.0337 (5)	
C11	0.5000	0.4876 (4)	0.4355 (2)	0.0403 (7)	
H11	0.5000	0.5931	0.4583	0.048*	
C12	0.5000	0.3596 (5)	0.4895 (2)	0.0476 (9)	
H12	0.5000	0.3772	0.5485	0.057*	
C13	0.5000	0.2045 (5)	0.4580 (3)	0.0503 (10)	
H13	0.5000	0.1138	0.4943	0.060*	
C14	0.5000	0.1873 (4)	0.3707 (3)	0.0462 (8)	
H14	0.5000	0.0832	0.3461	0.055*	
C15	0.5000	0.3219 (4)	0.3206 (2)	0.0382 (7)	
H15	0.5000	0.3084	0.2613	0.046*	
O1	0.40265 (11)	0.78003 (17)	0.34197 (9)	0.0387 (3)	
O2	0.40418 (10)	0.60368 (17)	0.19620 (9)	0.0367 (3)	
C2	0.26139 (19)	0.4840 (3)	0.14949 (17)	0.0528 (6)	
H2A	0.2535	0.5503	0.0990	0.079*	
H2B	0.1979	0.4547	0.1716	0.079*	
H2C	0.2972	0.3862	0.1352	0.079*	
C3	0.31578 (16)	0.5768 (3)	0.21467 (14)	0.0366 (5)	
C4	0.27229 (17)	0.6295 (3)	0.28825 (14)	0.0453 (6)	
H4	0.2081	0.5956	0.2990	0.054*	
C5	0.31605 (15)	0.7284 (3)	0.34724 (15)	0.0397 (5)	
C6	0.2633 (2)	0.7827 (4)	0.42460 (18)	0.0568 (7)	
H6A	0.3065	0.7769	0.4732	0.085*	
H6B	0.2074	0.7130	0.4341	0.085*	
H6C	0.2415	0.8938	0.4170	0.085*	

### Atomic displacement parameters (Å^2^)

**Table:**

	*U*^11^	*U*^22^	*U*^33^	*U*^12^	*U*^13^	*U*^23^
Mn1	0.0342 (2)	0.0313 (2)	0.0288 (2)	0.000	0.000	0.0002 (2)
S1	0.0976 (10)	0.0392 (5)	0.0406 (6)	0.000	0.000	0.0123 (4)
C1	0.0412 (16)	0.0363 (16)	0.0321 (15)	0.000	0.000	−0.0037 (12)
N1	0.0504 (16)	0.0389 (14)	0.0432 (17)	0.000	0.000	0.0150 (13)
N11	0.0446 (13)	0.0317 (12)	0.0249 (12)	0.000	0.000	−0.0003 (10)
C11	0.0493 (18)	0.0427 (16)	0.0289 (15)	0.000	0.000	−0.0039 (13)
C12	0.052 (2)	0.059 (2)	0.0315 (18)	0.000	0.000	0.0101 (15)
C13	0.050 (2)	0.052 (2)	0.049 (2)	0.000	0.000	0.0154 (18)
C14	0.0520 (19)	0.0347 (18)	0.052 (2)	0.000	0.000	0.0025 (15)
C15	0.0486 (17)	0.0352 (15)	0.0307 (15)	0.000	0.000	−0.0058 (12)
O1	0.0418 (8)	0.0360 (8)	0.0382 (8)	0.0033 (6)	0.0038 (7)	−0.0023 (7)
O2	0.0365 (8)	0.0418 (8)	0.0320 (8)	−0.0014 (6)	−0.0003 (6)	0.0017 (6)
C2	0.0488 (14)	0.0602 (16)	0.0496 (14)	−0.0110 (12)	−0.0093 (12)	−0.0002 (12)
C3	0.0347 (11)	0.0375 (10)	0.0376 (11)	−0.0009 (8)	−0.0052 (9)	0.0077 (9)
C4	0.0350 (11)	0.0573 (14)	0.0436 (15)	−0.0014 (10)	0.0030 (9)	0.0027 (10)
C5	0.0394 (11)	0.0416 (11)	0.0380 (11)	0.0085 (9)	0.0054 (10)	0.0038 (9)
C6	0.0515 (15)	0.0711 (19)	0.0478 (13)	0.0122 (13)	0.0116 (12)	−0.0034 (14)

### Geometric parameters (Å, º)

**Table:**

Mn1—O2	1.9185 (15)	C14—C15	1.374 (4)
Mn1—O2^i^	1.9185 (15)	C14—H14	0.9500
Mn1—O1^i^	1.9216 (15)	C15—H15	0.9500
Mn1—O1	1.9216 (15)	O1—C5	1.279 (3)
Mn1—N1	2.165 (3)	O2—C3	1.281 (3)
Mn1—N11	2.352 (2)	C2—C3	1.497 (3)
S1—C1	1.617 (3)	C2—H2A	0.9800
C1—N1	1.165 (4)	C2—H2B	0.9800
N11—C15	1.331 (4)	C2—H2C	0.9800
N11—C11	1.338 (4)	C3—C4	1.388 (3)
C11—C12	1.368 (5)	C4—C5	1.388 (3)
C11—H11	0.9500	C4—H4	0.9500
C12—C13	1.384 (6)	C5—C6	1.501 (3)
C12—H12	0.9500	C6—H6A	0.9800
C13—C14	1.396 (6)	C6—H6B	0.9800
C13—H13	0.9500	C6—H6C	0.9800
			
O2—Mn1—O2^i^	87.77 (9)	C15—C14—C13	119.5 (3)
O2—Mn1—O1^i^	174.74 (7)	C15—C14—H14	120.2
O2^i^—Mn1—O1^i^	91.20 (6)	C13—C14—H14	120.2
O2—Mn1—O1	91.20 (6)	N11—C15—C14	122.7 (3)
O2^i^—Mn1—O1	174.74 (7)	N11—C15—H15	118.7
O1^i^—Mn1—O1	89.36 (9)	C14—C15—H15	118.7
O2—Mn1—N1	92.46 (8)	C5—O1—Mn1	125.95 (15)
O2^i^—Mn1—N1	92.46 (8)	C3—O2—Mn1	127.15 (14)
O1^i^—Mn1—N1	92.74 (7)	C3—C2—H2A	109.5
O1—Mn1—N1	92.74 (7)	C3—C2—H2B	109.5
O2—Mn1—N11	89.47 (6)	H2A—C2—H2B	109.5
O2^i^—Mn1—N11	89.47 (6)	C3—C2—H2C	109.5
O1^i^—Mn1—N11	85.36 (6)	H2A—C2—H2C	109.5
O1—Mn1—N11	85.36 (6)	H2B—C2—H2C	109.5
N1—Mn1—N11	177.31 (11)	O2—C3—C4	123.7 (2)
N1—C1—S1	179.8 (3)	O2—C3—C2	114.5 (2)
C1—N1—Mn1	176.6 (3)	C4—C3—C2	121.8 (2)
C15—N11—C11	118.1 (3)	C5—C4—C3	124.5 (2)
C15—N11—Mn1	122.9 (2)	C5—C4—H4	117.7
C11—N11—Mn1	119.0 (2)	C3—C4—H4	117.7
N11—C11—C12	122.7 (3)	O1—C5—C4	124.5 (2)
N11—C11—H11	118.6	O1—C5—C6	114.3 (2)
C12—C11—H11	118.6	C4—C5—C6	121.3 (2)
C11—C12—C13	119.9 (3)	C5—C6—H6A	109.5
C11—C12—H12	120.0	C5—C6—H6B	109.5
C13—C12—H12	120.0	H6A—C6—H6B	109.5
C12—C13—C14	117.1 (4)	C5—C6—H6C	109.5
C12—C13—H13	121.5	H6A—C6—H6C	109.5
C14—C13—H13	121.5	H6B—C6—H6C	109.5

Symmetry code: (i) −*x*+1, *y*, *z*.
